# A Novel Purine and Uric Metabolism Signature Predicting the Prognosis of Hepatocellular Carcinoma

**DOI:** 10.3389/fgene.2022.942267

**Published:** 2022-07-12

**Authors:** Shengjie Yang, Baoying Zhang, Weijuan Tan, Lu Qi, Xiao Ma, Xinghe Wang

**Affiliations:** ^1^ Phase I Clinical Trial Center, Beijing Shijitan Hospital, Capital Medical University, Beijing, China; ^2^ Department of Internal Medicine, Zhangqiu People’s Hospital, Jinan, China

**Keywords:** hepatocellular carcinoma, prognosis, metabolism signature, purine, uric acid

## Abstract

**Background:** Hepatocellular carcinoma (HCC) is regarded as one of the most common cancers in the world with a poor prognosis. Patients with HCC often have abnormal purine and uric acid metabolism, but their relationship with prognosis is unclear.

**Methods:** Here, we collected the data of peripheral blood uric acid and clinical data in 50 patients with HCC and analyzed the relationship with prognosis. At the same time, the transcriptome sequencing data of TCGA and GEO databases were collected to analyze the changes in purine metabolic pathway activity and construct a prognosis prediction model. Based on the prognosis prediction model related to purine metabolism, we further looked for the differences in the immune microenvironment and molecular level and provided possible drug targets.

**Results:** We found that the level of serum uric acid was positively correlated with the prognosis of HCC. At the same time, purine metabolism and purine biosynthesis pathway activities were significantly activated in patients with a poor prognosis of HCC. The prognosis prediction model of HCC based on purine metabolism and purine biosynthesis pathway can accurately evaluate the prognosis of patients with HCC. Meanwhile, we found that there were significant changes in tumor immune infiltration microenvironment and biological function at the molecular level in patients with over-activation of purine metabolism and purine biosynthesis pathway. In addition, we found that uric acid level was positively correlated with peripheral blood leukocytes in HCC patients.

**Conclusion:** In this study, we found that the level of peripheral blood uric acid in patients with HCC is correlated with their prognosis. The prognosis of patients with HCC can be accurately predicted through the metabolic process of uric acid and purine.

## Introduction

Hepatocellular carcinoma (HCC) is the most common histological subtype of primary liver cancer, accounting for about 90% of all cases (Yuan et al., 2020). The malignant rate of HCC is very high, causing about 700,000 deaths every year in the world. It is the fourth leading cause of cancer death in the world (Lee and Lee, 2020). The existing treatment schemes include surgical resection, local ablation, liver transplantation, chemotherapy, molecular targeted therapy, and immunotherapy (Dai et al., 2022; Kong et al., 2022). However, due to the difficulty of early diagnosis of HCC and the high rate of tumor invasion and recurrence, the overall survival rate and prognosis of patients have been poor, and the average median survival time is about 1 year.

The imbalance of the tumor metabolic pathway is closely related to tumor growth and proliferation (Ke et al., 2022). Tumors need to increase energy consumption to maintain a high metabolic rate, which leads to the change of key metabolic pathways (Vasan and Cantley, 2022). The metabolic activity of tumor cells is significantly different from that of normal cells, and purine metabolism is an important part of material metabolism, which provides necessary components for DNA and RNA (Yuan et al., 2022). The genes of the purine synthesis pathway also have an impact in the field of tumors.

Studies have shown that the metabolic imbalance of purine and uric acid is one of the most significant metabolic changes in cancer, which not only affects the growth of primary tumors but also mediates tumor progression and metastasis (Wu et al., 2021). However, the relationship between purine and uric acid metabolism and the prognosis of patients with HCC has not been fully clarified.

In our study, we collected the data of peripheral blood uric acid in 50 patients with HCC and analyzed the relationship with prognosis. At the same time, the transcriptome sequencing data of TCGA and GEO databases were collected to analyze the changes in purine metabolic pathway activity and construct a prognosis prediction model. Based on the prognosis prediction model related to purine metabolism, we further looked for the differences in the immune microenvironment and molecular level and provided possible drug targets.

## Methods

### Data Collection of Hepatocellular Carcinoma Patients

The patient’s research was approved by the Beijing Shijitan Hospital and obtained the patient’s informed consent. A total of 50 patients with HCC were included in this study. Their gender, age, tumor stage, survival time, survival status, peripheral blood uric acid content, and peripheral blood leukocyte count were collected. See Supplementary Table S1 for details. The whole research process does not change the routine of clinical diagnosis and treatment, which belongs to observational research.

### Survival Analysis of HCC Patients With High Uric Acid and Low Uric Acid

First, according to the median uric acid content of 50 patients with HCC, they were divided into two groups: high UA and low UA, with 25 people in each group. The survival package of R software was used to analyze the KM survival of high uric acid (high UA) and low uric acid (low UA) groups in order to identify whether there are survival differences between high UA and low UA groups.

### Collection and Preprocessing of Transcriptome Data and Clinical Data

The transcriptome sequencing data and corresponding clinical information of HCC patients were downloaded from TCGA database (https://www.cancer.gov/about-nci/organization/ccg/research/structural-genomics/tcga). A total of 372 patients were collected as training sets. Two independent HCC patient data sets GSE54236 and GSE27150 were downloaded from the GEO database (https://www.ncbi.nlm.nih.gov) as validation sets. Transcriptome sequencing data and corresponding clinical information were collected.

### The Transcriptome Sequencing Data Were Analyzed for 113 Metabolic-Related Pathways and GSVA Function Score

The gene set of 113 metabolic-related pathways comes from published articles (Possemato et al., 2011). Through the R software GSVA package, 113 metabolic pathways of transcriptome sequencing data are scored for GSVA. The specific parameters are as follows: kcdf = “Poisson” and method = “gsva".

### Screening Metabolic Pathways Related to the Prognosis of HCC by Univariate Cox Regression

After integrating the GSVA score of 113 metabolic pathways with the prognosis of HCC, univariate Cox regression analysis was carried out by R software. *p* < 0.05 was selected as the statistical difference standard to screen the metabolic pathways related to survival.

### Differential Expression Gene Analysis

The differential analysis process was completed by the limma package. The transcriptome sequencing data after standardization were selected. The differential expression genes (DEGs) screening criteria were | log2FC ≥ 2| and *p* < 0.05.

### GO and KEGG Functional Enrichment Analysis

GO and KEGG function enrichment analysis is completed by g:Profiler online analysis website, and default parameters are selected for analysis (https://biit.cs.ut.ee/gprofiler/gost). The upregulated DEGs and downregulated DEGs were introduced into the g:Profiler online analysis website, respectively, and *p* < 0.05 was selected as the statistical difference threshold.

### Protein Interaction Analysis

PPI analysis is completed by STRING online analysis website (https://cn.string-db.org/), and the default parameters are selected for analysis. The DEGs were led to the STRING online analysis website to generate protein–protein interaction coefficients. The data were downloaded and imported into Cytoscape software to construct a protein–protein interaction network. The key modules are mined through the MCODE plug-in, and the key hub genes are found through the Cytohubber plug-in.

### Statistical Analysis

R software (version: 4.1.1) is used for statistical analysis and mapping. The information and survival time of patients were counted and plotted with Kaplan Meier curve. In the 3- and 5-year survival data, the prognostic correlation of genes was evaluated by distinguishing the area under the curve (AUC) generated by the curve. The Cox regression model was used for univariate analysis. An independent sample t-test was applied when only two groups were compared, whereas comparisons among groups were analyzed by two-way ANOVA. For all statistical methods, *p* < 0.05 was considered to be a significant difference.

## Results

### Uric Acid was Positively Correlated With Poor Prognosis in Patients With Hepatocellular Carcinoma

**FIGURE 1:**
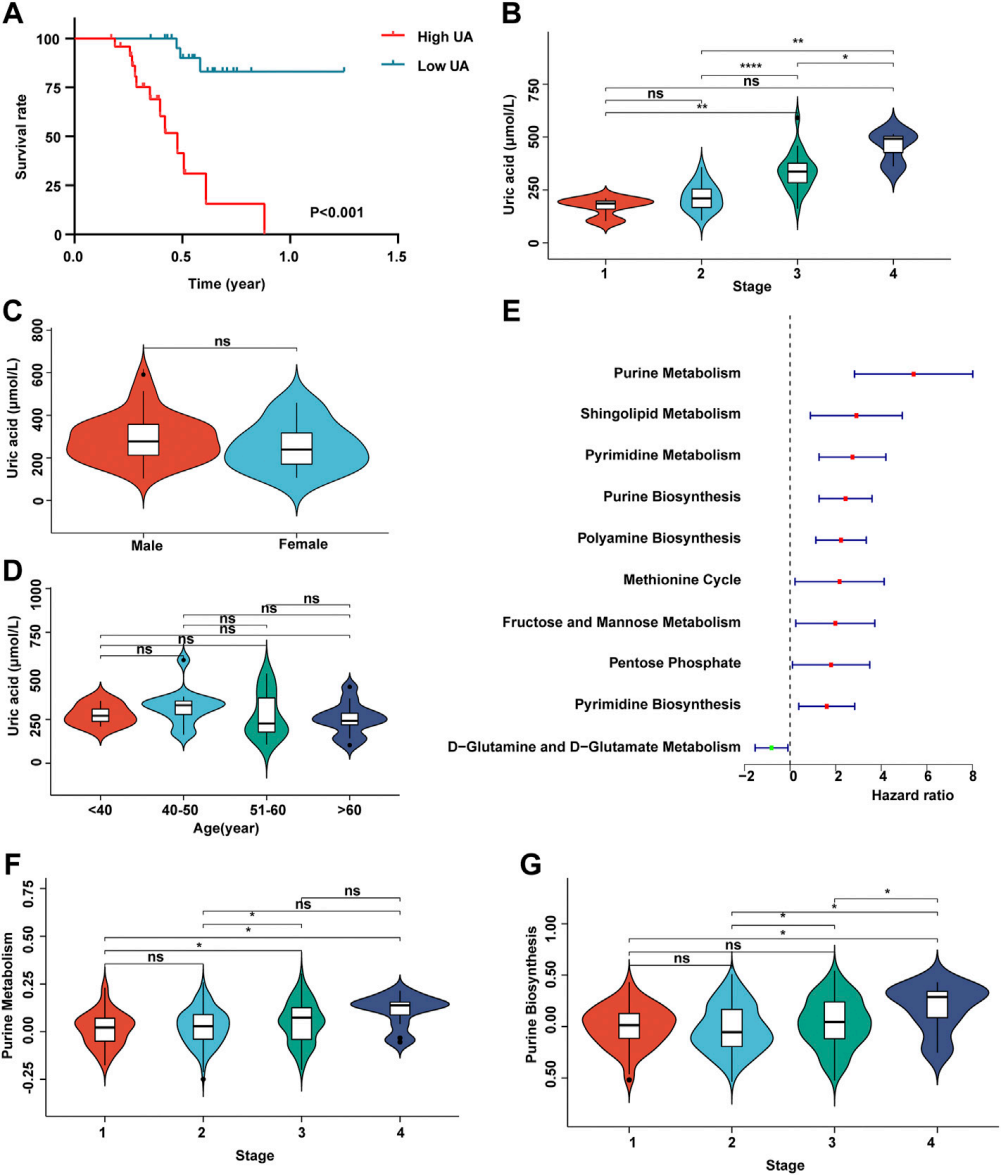
Uric acid was positively correlated with poor prognosis in patients with HCC. **(A)** Demonstrate the relationship between uric acid and survival curve; **(B)** content of uric acid in peripheral blood of patients with different stages of HCC; **(C)** content of uric acid in peripheral blood of patients with HCC in different genders; **(D)** content of uric acid in peripheral blood of patients with HCC at different ages; **(E)** forest map shows the relationship between different metabolic pathways and the prognosis of patients with HCC; **(F)** purine metabolic pathway activity in patients with different stages of HCC; **(G)** purine synthesis pathway activity in patients with different stages of HCC.

In order to detect whether uric acid-related metabolism is related to the prognosis of patients with HCC, we recruited 50 patients with HCC and collected clinical data such as uric acid in peripheral blood, tumor stage, survival time, gender, and age (Supplementary Table S1). Survival analysis showed that the higher the content of uric acid in peripheral blood, the worse the prognosis of patients with HCC (Figure 1A). Next, we analyzed the expression of uric acid in patients with different stages of HCC. The results shown in Figure 1B that the content of uric acid in peripheral blood in high tumor stages (stages 3 and 4) was significantly higher than that in low tumor stages (stages 1 and 2). At the same time, the content of uric acid was not related to gender and age (Figures 1C,D).

Subsequently, we collected the transcriptome expression data of 372 patients and their corresponding clinical information (including tumor stage, survival time, gender, and age) from TCGA database. First, we score 113 metabolic pathways through the GSVA algorithm (Supplementary Table S2). Then, we carried out a univariate Cox regression analysis of 113 metabolic-related pathways (Supplementary Table S3). The results showed that the metabolic pathways related to purine metabolism and purine synthesis were significantly correlated with the poor prognosis of patients with HCC (Figure 1E). The enhancement of purine metabolism can produce uric acid accumulation, which is one of the pathogeneses of gout. Meanwhile, purine metabolism and purine synthesis pathways had no correlation with tumor stage (Figures 1F,G).

### Construct a Prognostic Model of Purine-Related Metabolic Pathway in Patients With HCC

**FIGURE 2:**
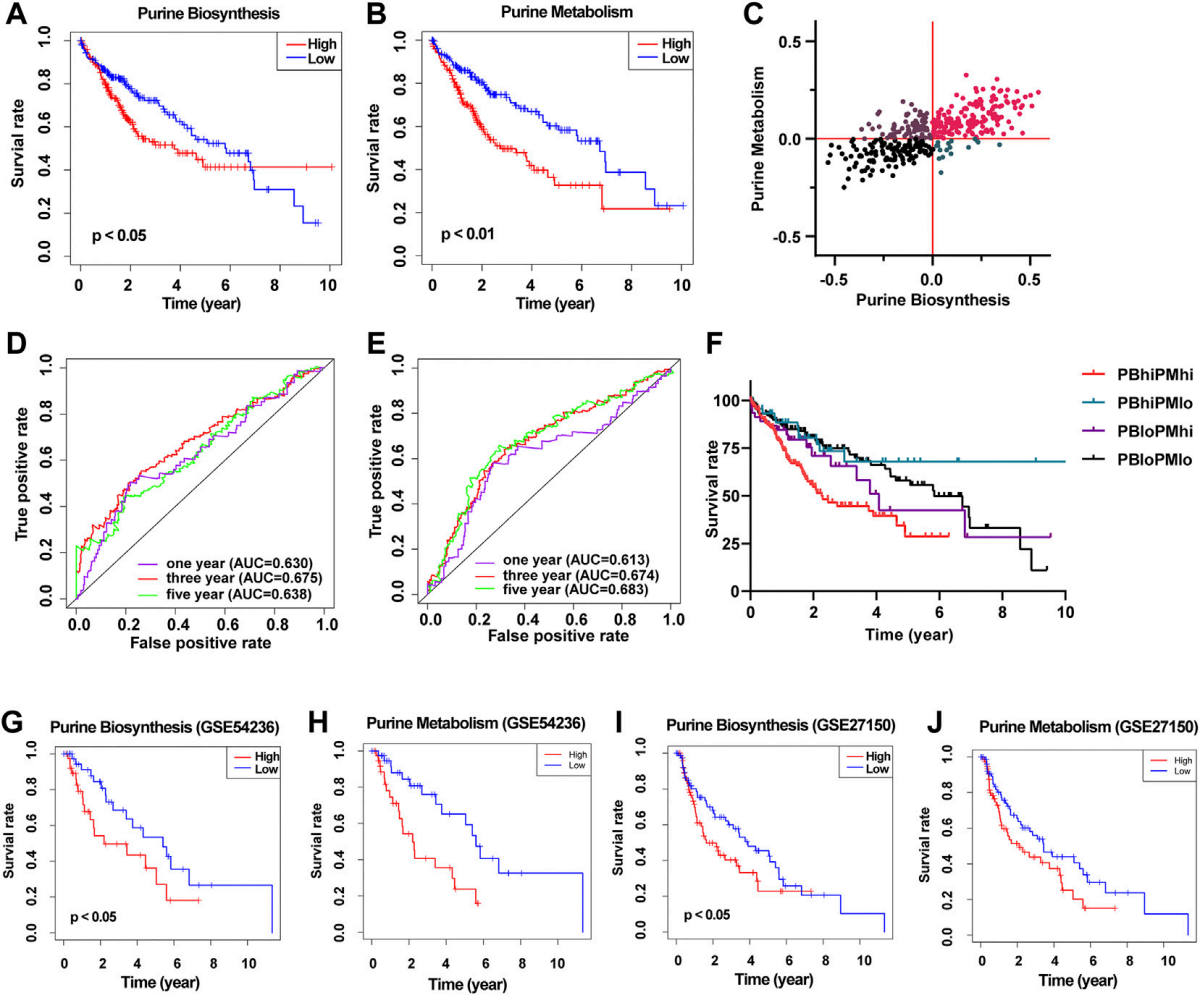
Construct a prognostic model of purine-related metabolic pathway in patients with HCC. **(A)** Survival curve showed the relationship between the activity of purine metabolism pathway and the prognosis of HCC; **(B)** survival curve showed the relationship between purine biosynthesis pathway activity and prognosis of HCC; **(C)** scatter plot shows the purine metabolic pathway activity and purine synthesis pathway activity of each patient with HCC and are divided into four groups according to the median of the two groups; **(D)** ROC curve shows the accuracy of predicting the prognosis of HCC according to the activity of purine metabolic pathway; **(E)** ROC curve shows the accuracy of predicting the prognosis of HCC according to the activity of purine synthesis pathway; **(F)** survival curve shows the prognosis of patients with HCC in different groups; **(G)** survival curve showed the relationship between the activity of purine synthesis pathway and the prognosis of HCC (GSE54236); **(H)** survival curve showed the relationship between purine metabolism pathway activity and prognosis of HCC (GSE54236); **(I)** survival curve showed the relationship between the activity of purine synthesis pathway and the prognosis of HCC (GSE27150); **(J)** survival curve showed the relationship between the activity of purine metabolism pathway and the prognosis of HCC (GSE27150).

Next, we divided the 371 HCC patients into the high score and low score groups according to the median GSVA score of purine metabolism and purine synthesis pathways. Survival analysis showed that the higher the GSVA score of purine metabolism and purine synthesis pathways, the worse the prognosis of patients with HCC (Figures 2A,B). At the same time, the ROC curve shows the accuracy of the prediction effect. The AUC values of 1, 3, and 5 years are greater than 0.6, showing good accuracy and specificity (Figures 2D,E). At the same time, we collected two independent data sets from the GEO database as verification sets, and the results are consistent with TCGA database (Figures 2F–J). In order to reduce the bias in the selection of purine metabolism gene sets, we collected another 11 purine metabolism-related gene sets from the GO database, KEGG database, Reactome database, and WikiPathways for verification. The results in 73% (8/11) of the gene sets were consistent with the previous results, and urine metabolism is a poor prognostic factor for HCC (Supplementary Table S4).—→

Then, according to the GSVA score of purine metabolism and purine synthesis pathways, we further divided HCC patients into four groups: high purine biosynthesis and high purine metabolism (PBhiPMhi), high purine biosynthesis and low purine metabolism (PBhiPMlo), low purine biosynthesis and high purine metabolism (PBloPMhi), and low purine biosynthesis and low purine metabolism (PBloPMlo) (Figure 2C). Survival analysis showed that the prognosis of PBhiPMhi was the worst, and the prognosis of the PBloPMlo group was the best, while the other two groups were in between (Figure 2F). These results suggest that purine metabolism and synthesis may be good predictors of prognosis.

### Purine Metabolism Affects Immune Infiltration Microenvironment in Patients With HCC

**FIGURE 3:**
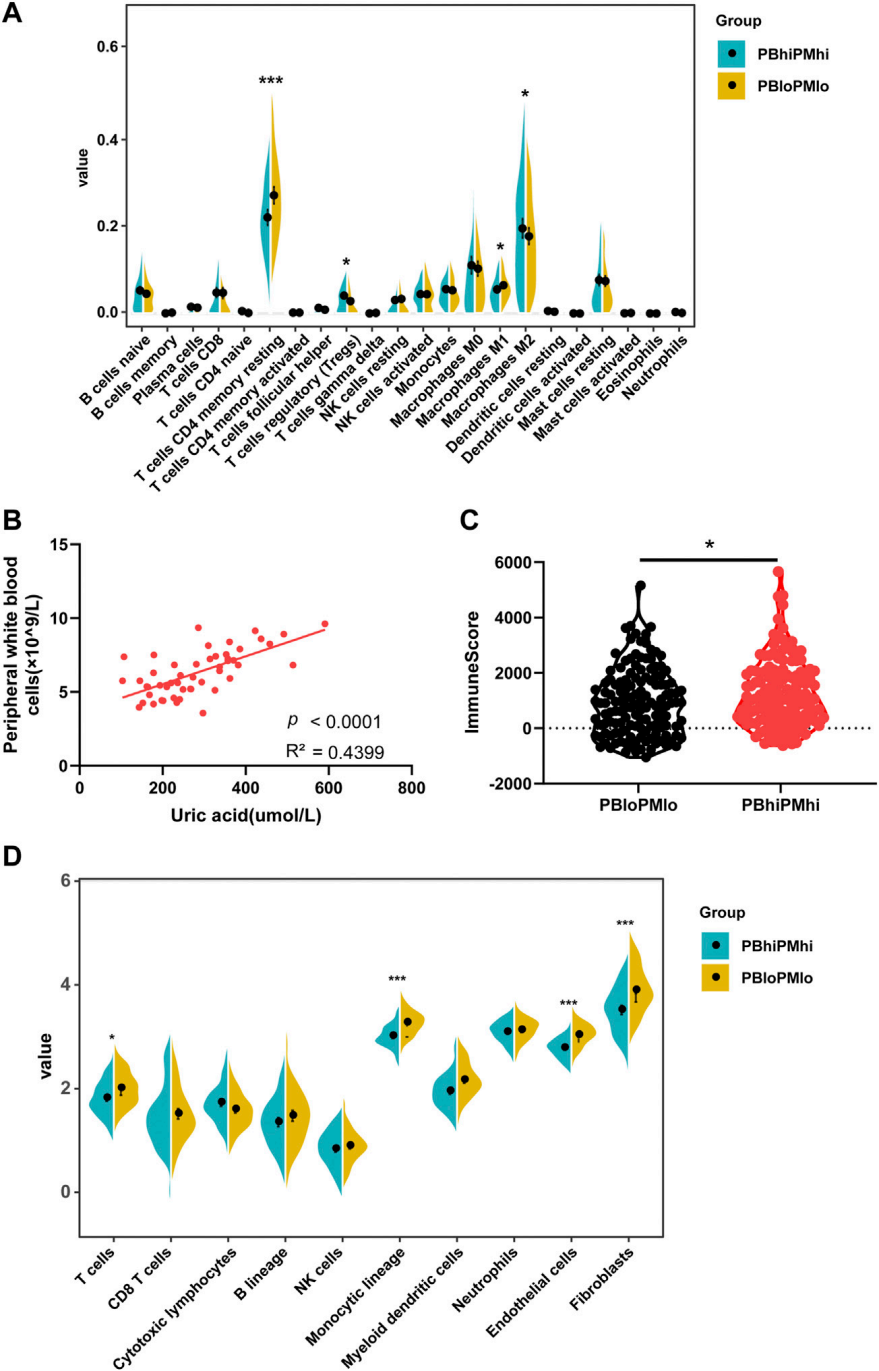
Purine metabolism affects immune infiltration microenvironment in patients with HCC. **(A)** Violin diagram shows the proportion of 22 immune cells in HCC patients in the PBhiPMhi group and PBloPMlo group. **(B)** Correlation between uric acid level and peripheral blood leukocytes in HCC patients. **(C–E)** Estimate and MCP count algorithms to verify the immune infiltration between PBloPMlo and PBhiPMhi groups.

The immune microenvironment is related to the prognosis of a variety of tumors. In order to explore whether purine-related metabolism can affect the tumor microenvironment of HCC patients, we analyzed 22 kinds of immune cells in the tumor immune infiltration microenvironment of 372 HCC patients in TCGA database by the CIBERSORT algorithm. The results of immune infiltration analysis showed that the contents of CD4^+^ T cells and M1 macrophages decreased significantly in patients with PBhiPMhi, while the contents of helper T cells and M2 macrophages increased significantly (Figure 3A). This suggests that purine-related metabolism is related to the immune infiltration microenvironment of the tumor. To be further explored the relationship between purine metabolism and immune cell infiltration, we collected peripheral blood leukocyte data from patients with HCC and found that uric acid level was positively correlated with peripheral blood leukocytes (Figure 3B). At the same time, in order to verify the reliability of CIBERSORT results, we also used MCP count and Estimate algorithms to verify the immune infiltration, and the results are basically consistent with the previous ones (Figures 3C,D).

### Analysis of Differential Gene Expressions Between PBhiPMhi and PBloPMlo Groups

**FIGURE 4:**
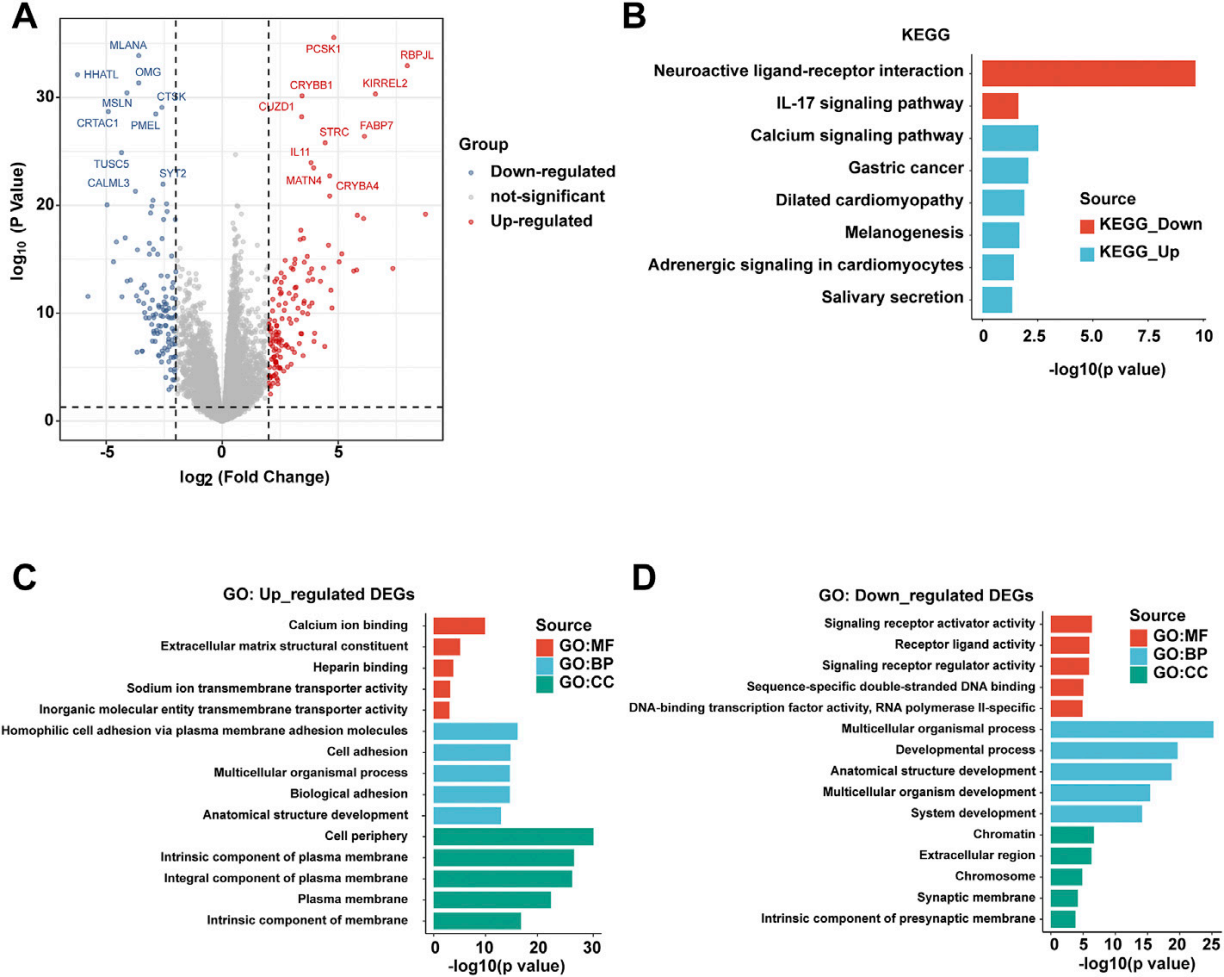
Analysis of differential gene expressions (DEGs) between PBhiPMhi and PBloPMlo groups. **(A)** Volcanic map shows the DEG distribution of HCC patients in the PBhiPMhi group and PBloPMlo group. **(B)** KEGG functional enrichment analysis of DEGs in patients with HCC; **(C,D)** GO functional enrichment analysis of DEGs in patients with HCC.

In order to further explore the differences at the molecular level between patients with PBhiPMhi and patients with PBloPMlo, we obtained the differential gene expressions (DEGs) between the two groups by the limma method. The screening criteria of DEGs were | log2fc ≥ 2 | and *p* < 0.05. The volcanic map showed the results of DEGs (Figure 4A). In order to further understand the function of these DEGs, we performed KEGG and GO functional enrichment analyses of upregulated DEGs and downregulated DEGs, respectively. KEGG enrichment analysis showed that the upregulated DEGs in the PBhiPMhi group were mainly enriched in neuroactive ligand receiver interaction and IL-17 signaling pathway, while the downregulated DEGs were enriched in calcium signaling pathway and gastric cancer (Figure 4B). GO enrichment analysis showed that the upregulated DEGs in the PBhiPMhi group were mainly enriched in the pathways related to the occurrence and development of cancer, while the downregulated DEGs were mainly enriched in the functions related to DNA damage repair (Figures 4C,D).

### Protein–Protein Interaction Analysis Predicts Possible Molecular Therapeutic Targets

**FIGURE 5:**
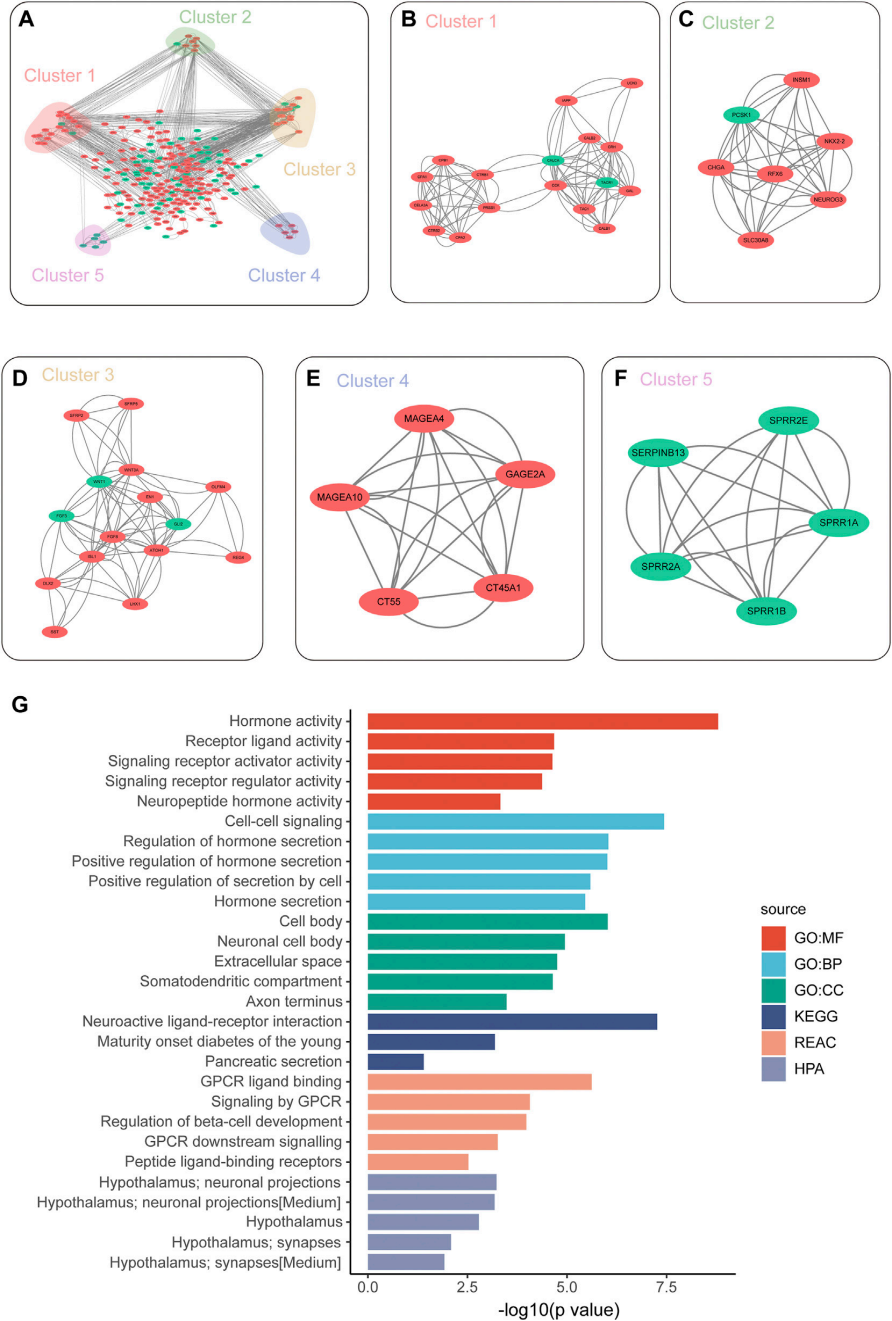
Protein–protein interaction (PPI) analysis predicts possible molecular therapeutic targets. **(A)** Network diagram shows the DEG protein–protein interaction network between the PBhiPMhi group and PBloPMlo group; **(B–F)** network diagram shows the five sub-networks of MCODE plug-in mining. **(G)** Functional enrichment analysis on these 20 hub genes in three databases.

In order to further find possible drug targets, we performed protein–protein interactions on different genes through the STRING database. Then, we can visualize the results in the database (Figure 5A). In order to find the key modules in the PPI network, we mine the modules of the PPI network through the MCODE plug-in. Finally, we found five key modules, which may be the key driver modules affecting the prognosis of HCC (Figures 5B–F). At the same time, we further explored the hub gene through the Cytohubber plug-in. We found a total of 20 key hub genes, such as recombinant somatostatin (SST), cholecystokinin (CCK), and tachykinin precursor 1 (TAC1). The functions of these genes need to be verified by experiments later (Supplementary Table S5). Then, we performed functional enrichment analysis on these 20 hub genes in three databases, and the results showed that those hub genes were mainly enriched in hormone activity, receptor-ligand activity, and GPCR ligand binding (Figure 5G).

## Discussion

Purine is a heterocyclic bicyclic aromatic organic molecule, which includes DNA, RNA, nucleosides and nucleotides, AMP, ADP, ATP, GMP, GDP, GTP, and cyclic forms of cAMP and cGMP, and participates in various metabolic pathways and cell signal transduction (La Grotta et al., 2022; Xiao et al., 2022). Uric acid is the product of purine metabolism. The enhancement of purine metabolism will lead to the accumulation of uric acid in peripheral blood. In this study, we collected the data of uric acid in peripheral blood of 50 patients with HCC, analyzed its relationship with the prognosis of patients with HCC, and analyzed the functional enrichment of transcriptome, immune microenvironment, KEGG, and GO in patients with HCC. Through comprehensive bioinformatics analysis, we established the prognosis prediction model of HCC and revealed the potential significance of the purine metabolic pathway in predicting the prognosis of HCC.

The imbalance of purine nucleotide metabolism often occurs in the process of tumor occurrence and development (Wang X. et al., 2022). Many enzymes involved in purine nucleotide anabolism and catabolism are related to the proliferation and drug resistance of tumor cells (Wu et al., 2022). The imbalance of antioxidant and pro-inflammatory properties of uric acid can also induce tumors and promote their progress (Wang J. et al., 2022). Abnormal purine nucleotide metabolism can affect the expression of genes and proteins by regulating the signal transduction pathway and promote cell malignant transformation, invasion, and metastasis (Yang et al., 2019). The characteristics of nucleotide metabolism are different in different tumor patients.

A large number of studies have found that the level of serum uric acid is positively correlated with the occurrence of colorectal cancer, liver cancer, kidney cancer, melanoma, and head and neck tumors, but its relationship with the prognosis of HCC is still unclear (Ayoub et al., 2021; Wang H. et al., 2022; Wang J. et al., 2022). In our study, we found that the level of serum uric acid was positively correlated with the prognosis of HCC, suggesting that serum uric acid may be used as an index to predict the prognosis of patients with HCC. At the same time, we found that purine metabolism and purine anabolic pathway activities were significantly activated in patients with poor prognoses of HCC. The prognosis prediction model of HCC based on purine metabolism and purine synthesis pathways can accurately evaluate the prognosis of patients with HCC. At the same time, we found that there were significant changes in tumor immune infiltration microenvironment and biological function at the molecular level in patients with over-activation of purine metabolism and purine synthesis pathways, suggesting that purine metabolism and purine synthesis can participate in multiple processes mediating tumor occurrence and development.

In order to further explore possible drug targets, we performed protein–protein interaction on different genes through the STRING database and found a total of 20 key hub genes based on purine metabolism and purine synthesis prediction model, such as SST, CCK, and TAC1. These hub genes may be potential therapeutic targets for HCC. Some studies showed that SST expression can be used to predict an unfavorable prognosis in patients with HCC (Kaemmerer et al., 2017; Murakami et al., 2021). Serum levels of CCK are elevated in HCC patients, and CCK blockade is a novel approach for the prevention/treatment of HCC. (Tucker et al., 2020; Gay et al., 2021). However, there are few studies on the relationship between TACR1 and HCC. In studies of other cancers, researchers found that TACR1 correlates with the prognosis of colorectal cancer (CRC) and gastric cancer (GC) (David et al., 2009; Yu et al., 2012). These targets may have to guide significance for the future study of HCC.

In conclusion, in this study, we found that the level of peripheral blood uric acid in patients with HCC is correlated with their prognosis. The prognosis of patients with HCC can be accurately predicted through the metabolic process of uric acid and purine. At the same time, based on the prediction model, we found the cellular and molecular mechanisms that may affect the prognosis of HCC, including tumor immune infiltration microenvironment, a variety of biological processes, and potential drug targets. In addition, we found that uric acid level was positively correlated with peripheral blood leukocytes in HCC patients. The limitation of this study is that it does not apply the data with more samples for large-scale verification. We will conduct relevant experiments in the future. In a word, these findings may provide a new way to predict the prognosis of HCC.

## Data Availability

The original contributions presented in the study are included in the article/Supplementary Material; further inquiries can be directed to the corresponding author.

## References

[B1] Ayoub A. M. Amin M. U. Ambreen G. Dayyih A. A. Abdelsalam A. M. Somaida A. (2022). Photodynamic and Antiangiogenic Activities of Parietin Liposomes in Triple Negative Breast Cancer. Biomater. Adv. 134, 112543. 10.1016/j.msec.2021.112543 35523642

[B2] Dai K. Liu C. Guan G. Cai J. Wu L. (2022). Identification of Immune Infiltration-Related Genes as Prognostic Indicators for Hepatocellular Carcinoma. BMC Cancer 22 (1), 496. 10.1186/s12885-022-09587-0 35513781 PMC9074323

[B3] David S. Kan T. Cheng Y. Agarwal R. Jin Z. Mori Y. (2009). Aberrant Silencing of the Endocrine Peptide Gene Tachykinin-1 in Gastric Cancer. Biochem. Biophysical Res. Commun. 378 (3), 605–609. 10.1016/j.bbrc.2008.11.078 19046942

[B4] Gay M. D. Safronenka A. Cao H. Liu F. H. Malchiodi Z. X. Tucker R. D. (2021). Targeting the Cholecystokinin Receptor: A Novel Approach for Treatment and Prevention of Hepatocellular Cancer. Cancer Prev. Res. (Phila) 14 (1), 17–30. 10.1158/1940-6207.Capr-20-0220 33115780 PMC8079543

[B5] Kaemmerer D. Schindler R. Mußbach F. Dahmen U. Altendorf-Hofmann A. Dirsch O. (2017). Somatostatin and CXCR4 Chemokine Receptor Expression in Hepatocellular and Cholangiocellular Carcinomas: Tumor Capillaries as Promising Targets. BMC Cancer 17 (1), 896. 10.1186/s12885-017-3911-3 29282035 PMC5745780

[B6] Ke X. Wu H. Chen Y.-X. Guo Y. Yao S. Guo M.-R. (2022). Individualized Pathway Activity Algorithm Identifies Oncogenic Pathways in Pan-Cancer Analysis. EBioMedicine 79, 104014. 10.1016/j.ebiom.2022.104014 35487057 PMC9117264

[B7] Kong W. Mao Z. Han C. Ding Z. Yuan Q. Zhang G. (2022). A Novel Epithelial-Mesenchymal Transition Gene Signature Correlated with Prognosis, and Immune Infiltration in Hepatocellular Carcinoma. Front. Pharmacol. 13, 863750. 10.3389/fphar.2022.863750 35517787 PMC9065556

[B8] La Grotta R. de Candia P. Olivieri F. Matacchione G. Giuliani A. Rippo M. R. (2022). Anti-inflammatory Effect of SGLT-2 Inhibitors via Uric Acid and Insulin. Cell. Mol. Life Sci. 79 (5), 273. 10.1007/s00018-022-04289-z 35503137 PMC9064844

[B9] Lee A. Lee F.-C. (2020). Medical Oncology Management of Advanced Hepatocellular Carcinoma 2019: a Reality Check. Front. Med. 14 (3), 273–283. 10.1007/s11684-019-0728-2 31863306

[B10] Murakami K. Kumata H. Miyagi S. Kamei T. Sasano H. (2021). The Prognostic Significance of Neuroendocrine Markers and Somatostatin Receptor 2 in Hepatocellular Carcinoma. Pathol. Int. 71 (10), 682–691. 10.1111/pin.13149 34320691

[B11] Possemato R. Marks K. M. Shaul Y. D. Pacold M. E. Kim D. Birsoy K. (2011). Functional Genomics Reveal that the Serine Synthesis Pathway Is Essential in Breast Cancer. Nature 476 (7360), 346–350. 10.1038/nature10350 21760589 PMC3353325

[B12] Tucker R. D. Ciofoaia V. Nadella S. Gay M. D. Cao H. Huber M. (2020). A Cholecystokinin Receptor Antagonist Halts Nonalcoholic Steatohepatitis and Prevents Hepatocellular Carcinoma. Dig. Dis. Sci. 65 (1), 189–203. 10.1007/s10620-019-05722-3 31297627 PMC6946881

[B13] Vasan N. Cantley L. C. (2022). At a Crossroads: How to Translate the Roles of PI3K in Oncogenic and Metabolic Signalling into Improvements in Cancer Therapy. Nat. Rev. Clin. Oncol. 19 (7), 471–485. 10.1038/s41571-022-00633-1 35484287 PMC11215755

[B14] Wang H. Xie L. Song X. Wang J. Li X. Lin Z. (2022a). Purine-Induced IFN-γ Promotes Uric Acid Production by Upregulating Xanthine Oxidoreductase Expression. Front. Immunol. 13, 773001. 10.3389/fimmu.2022.773001 35154100 PMC8829549

[B15] Wang J. Liu K. Xiao T. Liu P. Prinz R. A. Xu X. (2022b). Uric Acid Accumulation in DNA-Damaged Tumor Cells Induces NKG2D Ligand Expression and Antitumor Immunity by Activating TGF-β-Activated Kinase 1. Oncoimmunology 11 (1), 2016159. 10.1080/2162402X.2021.2016159 35154904 PMC8837239

[B16] Wang X. Su W. Jiang Y. Jia F. Huang W. Zhang J. (2022c). Regulation of Nucleotide Metabolism with Nutrient‐Sensing Nanodrugs for Cancer Therapy. Adv. Sci., 2200482. 10.1002/advs.202200482 PMC9284143 35508896

[B17] Wu H.-l. Gong Y. Ji P. Xie Y.-f. Jiang Y.-Z. Liu G.-y. (2022). Targeting Nucleotide Metabolism: a Promising Approach to Enhance Cancer Immunotherapy. J. Hematol. Oncol. 15 (1), 45. 10.1186/s13045-022-01263-x 35477416 PMC9044757

[B18] Wu L. Yang W. Zhang Y. Du X. Jin N. Chen W. (2021). Elevated Serum Uric Acid Is Associated with Poor Survival in Advanced HCC Patients and Febuxostat Improves Prognosis in HCC Rats. Front. Pharmacol. 12, 778890. 10.3389/fphar.2021.778890 34858193 PMC8632057

[B19] Xiao L. Sha W. Tao C. Hou C. Xiao G. Ren J. (2022). Effect on Purine Releasement of Lentinus Edodes by Different Food Processing Techniques. Food Chem. X 13, 100260. 10.1016/j.fochx.2022.100260 35498996 PMC9040045

[B20] Yang S. He X. Liu Y. Ding X. Jiang H. Tan Y. (2019). Prognostic Significance of Serum Uric Acid and Gamma-Glutamyltransferase in Patients with Advanced Gastric Cancer. Dis. Markers 2019, 12. 10.1155/2019/1415421 PMC6918938 31885729

[B21] Yu Y. Pan Y. Jin M. Zhang M. Zhang S. Li Q. (2012). Association of Genetic Variants in Tachykinins Pathway Genes with Colorectal Cancer Risk. Int. J. Colorectal Dis. 27 (11), 1429–1436. 10.1007/s00384-012-1478-7 22733436

[B22] Yuan K. Xie K. Lan T. Xu L. Chen X. Li X. (2020). TXNDC12 Promotes EMT and Metastasis of Hepatocellular Carcinoma Cells via Activation of β-catenin. Cell. Death Differ. 27 (4), 1355–1368. 10.1038/s41418-019-0421-7 31570854 PMC7206186

[B23] Yuan R.-H. Hsu C.-L. Jhuang Y.-L. Liu Y.-R. Hsieh T.-H. Jeng Y.-M. (2022). Tumor-matrix Interaction Induces Phenotypic Switching in Liver Cancer Cells. Hepatol. Int. 16, 562–576. 10.1007/s12072-022-10315-w 35525880

